# Chronic Invasive Nongranulomatous Fungal Rhinosinusitis in Immunocompetent Individuals

**DOI:** 10.1155/2016/6854121

**Published:** 2016-09-15

**Authors:** Ozge Turhan, Asli Bostanci, Irem Hicran Ozbudak, Murat Turhan

**Affiliations:** ^1^Department of Infectious Diseases, Akdeniz University School of Medicine, Antalya, Turkey; ^2^Department of Otolaryngology, Head and Neck Surgery, Akdeniz University School of Medicine, Antalya, Turkey; ^3^Department of Pathology, Akdeniz University School of Medicine, Antalya, Turkey

## Abstract

Chronic invasive nongranulomatous fungal rhinosinusitis is a well-described but uncommon type of fungal rhinosinusitis (FRS). While the prevalence of chronic FRS is 0.11% in healthy individuals, only 1.3% of them are in nongranulomatous invasive nature. The majority of the cases in the literature have been reported from developing countries mostly located in the tropical regions, as typically occurring in the background of diabetes mellitus or corticosteroid treatment. The current paper reports four consecutive cases, who were diagnosed within a short period of six months at a single center of a country located outside the tropical climate zone. None of the patients had a comorbid disease that may cause immune suppression or a history of drug use. The only risk factor that may have a role in development of chronic invasive nongranulomatous FRS was that all of our patients were people working in greenhouse farming. Three cases underwent endoscopic sinus surgery, and one case underwent surgery with both endoscopic and external approaches. Systemic antifungal therapy was initiated in all cases in the postoperative period with voriconazole 200 mg orally twice a day. All patients achieved a complete clinical remission. Chronic invasive nongranulomatous FRS should be kept in mind in the presence of long-standing nonspecific sinonasal symptoms in immunocompetent individuals, particularly with a history of working in greenhouse farming.

## 1. Introduction

Fungal rhinosinusitis (FRS) encompasses a spectrum of sinonasal diseases with distinct clinical courses, histopathologies, and disease outcomes. FRS is classified into two groups as invasive and noninvasive depending on invasion of the mucosal layer by fungi. Noninvasive FRS includes saprophytic fungal infestation, fungal ball, and allergic FRS. Invasive FRS is subdivided into acute invasive, chronic nongranulomatous invasive, and chronic granulomatous types [1].

Chronic nongranulomatous invasive FRS is a well-described but uncommon type of FRS. While the prevalence of chronic FRS is 0.11% in healthy individuals, only 1.3% of them are in nongranulomatous invasive nature [2]. In this paper, four consecutive immunocompetent patients with chronic invasive nongranulomatous FRS, who were diagnosed over a period of 6 months at a single center (June 2015–October 2015), were presented along with the literature.

## 2. Case Presentation

The current study was conducted in accordance with the Declaration of Helsinki and with approval from the Institutional Ethics Committee. Written informed consent was obtained from the patients.

All patients were farmers from rural areas. A comprehensive head and neck examination was performed in all cases. Complete blood cell counts and serum chemistry panel, including hepatic and renal function tests, were evaluated. Radiological evaluation was carried out by computed tomography (CT) scan. None of the patients had a comorbid disease that may cause immune suppression or a history of drug use. Three cases underwent endoscopic sinus surgery, and one case underwent sinus surgery with both endoscopic and external approaches. A Gomori methenamine-silver stain was used in the histopathological diagnosis. The diagnosis of chronic invasive FRS was made by the demonstration of silver accumulation in the fungal cell wall, the presence of hyphal forms within the submucosa, and the demonstration of tissue necrosis accompanied by minimal host inflammatory cell infiltration [3]. It was distinguished from granulomatous FRS by dense accumulation of hyphae, occasional angioinvasion, sparse inflammatory infiltrate, and lack of submucosal granulomatous inflammation containing giant cells [4]. Histopathological diagnosis was confirmed by fungal culture, although it was not an absolute requirement [5].

Systemic antifungal therapy was initiated in all cases in the postoperative period with voriconazole 200 mg orally twice a day. The duration of treatment was decided at the discretion of the institutional local committee on infectious diseases. Although the treatment was maintained for six weeks in three cases, it was terminated in the third week in one case due to the adverse effects. Patients were followed up for recurrence once every three months by physical and endoscopic examinations. Demographic, clinical, and radiological data were recorded for all patients (Table 1).

### 2.1. Case 1

**Figure 1:**
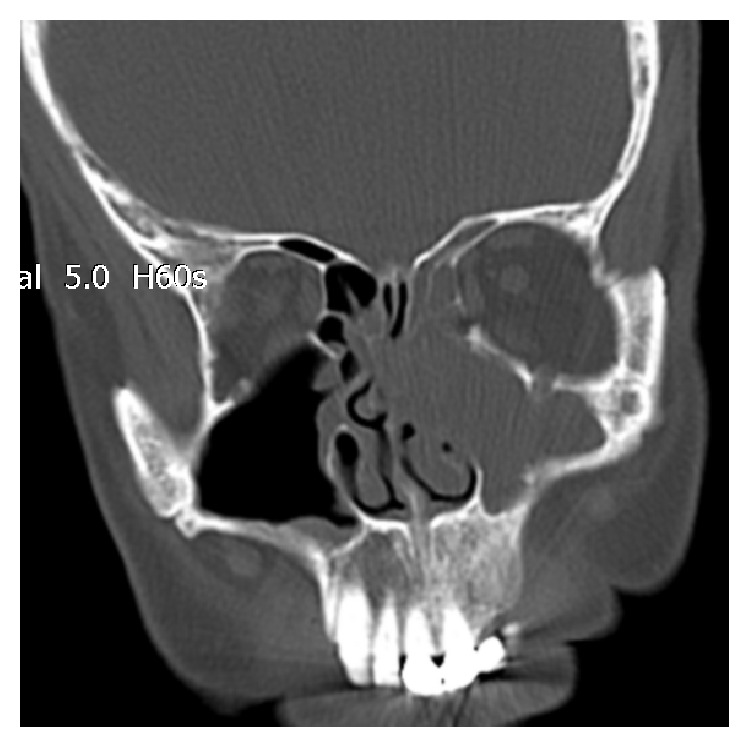
Paranasal sinus computed tomography showing a soft tissue lesion eroding the left maxillary sinus medial wall, filling the ethmoidal cells and frontal sinus and destructing the lamina papyracea.

A 58-year-old male patient was admitted with a 6-month history of nasal obstruction, swelling on the left side of the face, and left orbital pain. Endoscopic examination revealed a polypoid mass and intense purulent secretion in the left nasal cavity and middle meatus. On paranasal sinus CT, an expansive soft tissue lesion that erodes the left maxillary sinus medial wall, fills the ethmoidal cells and frontal sinus, and leads to the destruction of lamina papyracea was observed (Figure 1). A gray-white, cheesy material was completely removed from all sinuses by endoscopic sinus surgery. Tissue culture identified the fungus as* Aspergillus fumigatus.* No recurrence was detected during a follow-up period of 10 months after systemic antifungal therapy was discontinued.

### 2.2. Case 2

**Figure 2:**
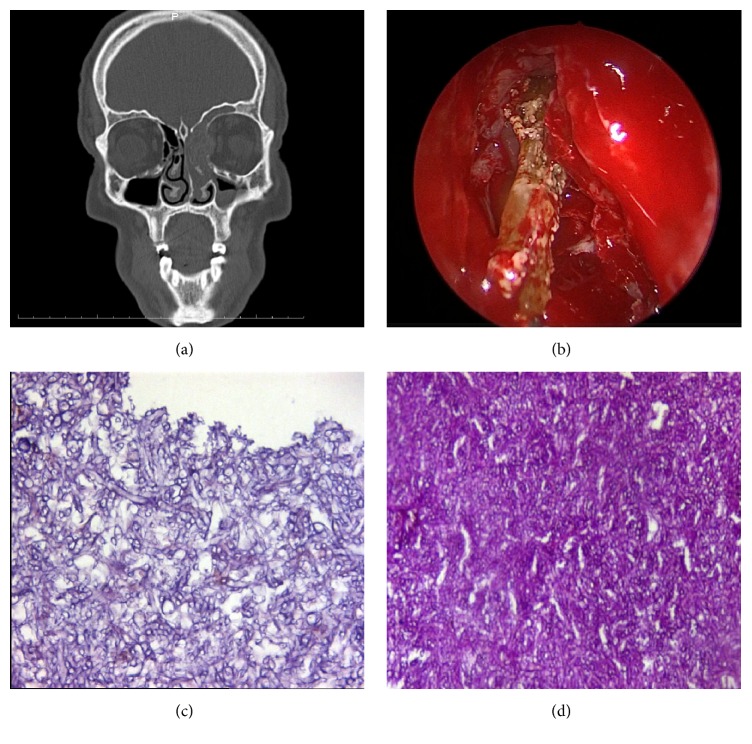
(a) Paranasal sinus computed tomography showing a soft tissue mass filling the left nasal cavity and ethmoid, frontal, and sphenoid sinuses and indenting the medial rectus muscle by destructing the lamina papyracea. (b) Endoscopic view of the yellow-green colored fungal debris. (c) Hematoxylin and eosin stain of the tissue revealing a group of narrow branching septate hyphae (×400). (d) Periodic acid-Schiff stain of the tissue showing branching septate hyphae.

A 43-year-old male patient was referred to our clinic with the complaints of nasal obstruction, headache, and facial pain which existed for about two years. It was learned that he had received multiple medications for rhinosinusitis without improvement of his symptoms. Endoscopic examination showed intense purulent discharge in the left nasal cavity and middle meatus. Paranasal sinus CT scan revealed a soft tissue mass that fills the left nasal cavity and ethmoid, frontal, and sphenoid sinuses and indents the medial rectus muscle by destructing the lamina papyracea (Figure 2(a)). Ophthalmologic examination was unremarkable. Left maxillary, ethmoid, frontal, and sphenoid sinuses were opened endoscopically, and a yellow-green gelatinous material was drained (Figure 2(b)). The left orbital medial wall was observed to be eroded, and the orbital adipose tissue was found to be exposed. An external surgical approach was performed to clear the tissues located at the most lateral part of the left frontal sinus. Histopathological examination was consistent with chronic nongranulomatous invasive FRS (Figures 2(c)-2(d)), while* Aspergillus flavus* was isolated in the culture. No recurrence occurred during a follow-up period of eight months, although the antifungal therapy was stopped on postoperative day 21 due to the adverse effects including blurred vision, vision color changes, and skin rashes.

### 2.3. Case 3

**Figure 3:**
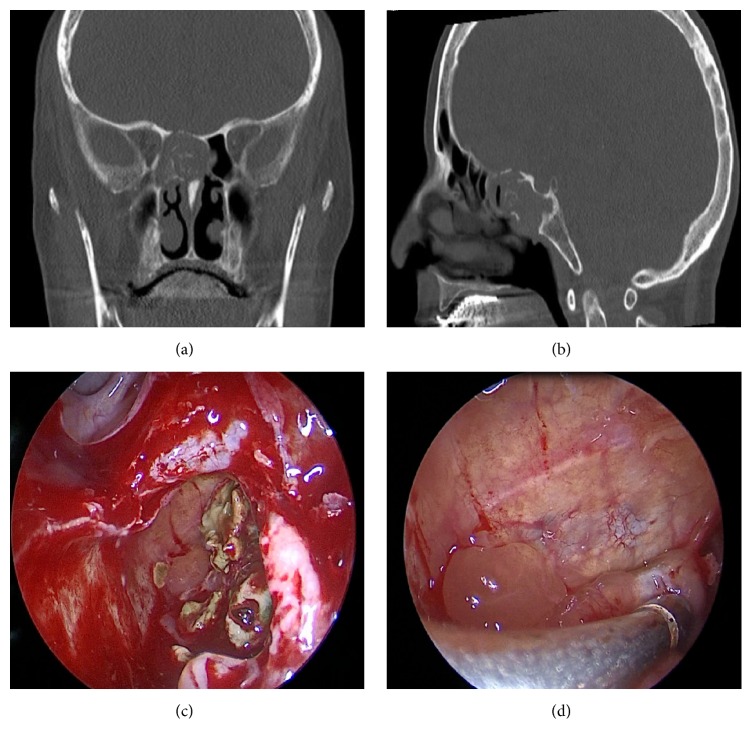
(a, b) Paranasal sinus computed tomography of a soft tissue mass filling the right posterior ethmoidal cells and sphenoid sinus. (c) Endoscopic view of the yellow-green colored caseous material in the right sphenoid sinus. (d) Endoscopic view of the intact dura mater in the right sphenoid sinus.

A 60-year-old female patient presented with a history of dizziness, headache, and postnasal drainage for nine months. Endoscopic examination of the nasal cavity and osteomeatal complex was unremarkable. A tattletale gray cheesy material was observed in the sphenoethmoidal recess. Paranasal sinus CT scan showed a soft tissue mass that fills the right posterior ethmoidal cells and sphenoid sinus and erodes the posterior wall of the sphenoid sinus and floor of the sella (Figures 3(a)-3(b)). She underwent total sphenoethmoidectomy through transnasal and transethmoidal approaches. Sphenoid sinus was fully filled by a yellow-green caseous material (Figure 3(c)). Fungal debris and underlying hypertrophic mucosa were excised. The floor of the sella was eroded, but the dura was intact (Figure 3(d)). Fungal culture was positive for* Aspergillus fumigatus*. The patient is disease-free at seven months after antifungal therapy.

### 2.4. Case 4

**Figure 4:**
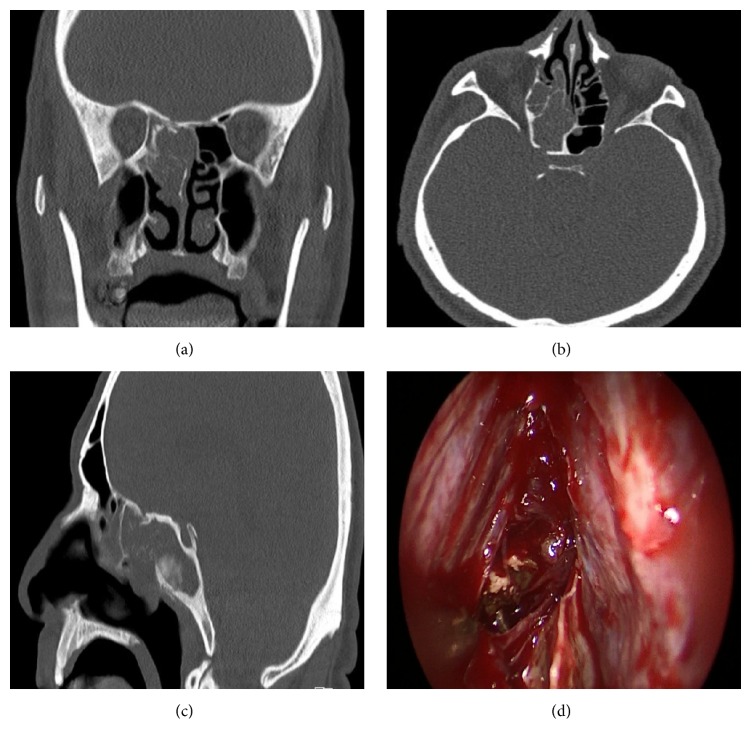
(a, b, and c) Paranasal sinus computed tomography of a hypodense mass filling the right sphenoid sinus (a-coronal, b-axial c-sagittal planes). (d) Endoscopic view of the caseous material filling the right sphenoid sinus.

A 68-year-old male patient presented with a history of nasal obstruction, headache, and left retroorbital pain for six months. Nasal endoscopy revealed drainage of purulent secretion from the right sphenoid sinus ostium. Paranasal sinus CT showed a hypodense mass filling the right sphenoid sinus (Figures 4(a)–4(c)). The patient underwent total sphenoethmoidectomy, and the caseous material within the sphenoid sinus was completely removed (Figure 4(d)). While the histopathological examination was consistent with chronic nongranulomatous invasive FRS, no growth was evident in the fungal culture. Six months postoperatively, he is free of symptoms and is doing well.

## 3. Discussion

Rhinosinusitis is a common public health problem that affects approximately 20% of the population [2]. While viruses and bacteria are the infectious agents detected in the majority of cases, fungi are responsible for certain particular subtypes [6].

Acute invasive FRS is an opportunistic fungal infection almost exclusively affecting immunocompromised individuals. Classical hosts include those receiving multiagent chemotherapy for a hematologic malignancy and those who are neutropenic or those with aplastic anemia [7]. The disease manifests with an acute onset and has a fulminant course. In a case series of 29 patients, Monroe et al. reported a six-month overall survival of only 18% [8].

In contrast to acute invasive disease, chronic forms of invasive FRS including granulomatous and nongranulomatous types are mostly seen in apparently healthy individuals [2]. Although these two forms of chronic invasive FRS are considered separate entities, they share many clinical and pathologic features.* Aspergillus* species are the most common fungi isolated in both forms. Tissue invasion by fungi occurs over a prolonged period (several weeks or months), rather than hours as in acute invasive FRS [7]. Patients often present with nonspecific symptoms and have an indolent clinical course. Therefore, they are usually associated with delayed diagnosis, which may increase the morbidity as well as mortality [7]. Despite the lack of comparative data, no significant differences in disease outcomes have been documented [1]. Both forms are treated in a similar fashion [5].

The distinction between granulomatous and nongranulomatous types of chronic invasive FRS is primarily based on pathological findings [1]. Granulomatous type is characterized by the presence of submucosal noncaseating granuloma consisting of foreign body or Langhans-type giant cells. Fungal hyphae are usually sparse, and there is extensive fibrosis. In contrast, there is a dense accumulation of hyphae in nongranulomatous type but the inflammatory infiltrate is sparse [4].

The majority of chronic invasive FRS cases in the literature have been reported from developing countries mostly located in the tropical regions of South Asia, Middle East, Africa, and South America [9]. The hot and humid climate in these regions creates a suitable environment for fungal growth and proliferation of spores. In addition, the high number of people working in agricultural activities increases the risk of intense exposure to fungal spores and development of fungal infection in a healthy individual. However, most of these reported cases include examples of granulomatous type.

Available data regarding chronic nongranulomatous invasive FRS are relatively sparse as compared to acute invasive and chronic granulomatous FRS. In most instances, the disease occurs in the background of diabetes mellitus or prolonged corticosteroid treatment [1]. In a multi-institutional analysis including 18 patients with invasive FRS (of them 8 were chronically nongranulomatous and 10 were acutely invasive), Pagella et al. reported a disease-related mortality rate of 25% for chronic invasive nongranulomatous FRS compared with 40% for acute invasive disease [10]. The majority of patients with chronic invasive disease had diabetes mellitus (87.5%) as comorbidity. On the other hand, in a small case series of 6 patients with chronic nongranulomatous invasive FRS, D'Anza et al. reported that all patients were free of disease at last follow-up, with a range of 1 to 27 months [11]. In this series, also all patients had systemic comorbidities, with diabetes mellitus being the most common.

In contrast to previous reports, none of the patients in our series had any comorbidities or history of corticosteroid use that may alter immune functions. In addition, our country remains outside the tropical climate zone. Apparently, the only risk factors in our patients were that they were rural and were working in greenhouse farming and the fact that four cases are being reported from a single center within a short period of six months is clinically important.

The treatment of invasive FRS, including both acute and chronic subtypes, requires an effective surgical debridement and systemic antifungal therapy [5]. The aim of the surgery is to provide adequate sinus ventilation by the removal of devitalized tissues and to facilitate the penetration of antifungal agents. Amphotericin B, voriconazole, itraconazole, posaconazole, and caspofungin are antifungal agents that are effective against* Aspergillus* species [12]. In the single randomized controlled trial comparing voriconazole with amphotericin B, voriconazole was associated with better response and survival rates and fewer side effects than amphotericin B [13]. The duration of antifungal therapy has not been optimally defined in clinical practice guidelines. On the other hand, immune status of the patient, extent of the disease, and stabilization of all clinical and radiographic manifestations have been proposed to be the main factors in decision making for duration of treatment [12]. An important issue that should be considered in the use of voriconazole is that it may lead to transient visual disturbances (44%) [13]. In one of the cases in our series, a partial visual impairment occurred in the third week of the treatment; however it resolved spontaneously within 72 hours after stopping voriconazole.

In conclusion, chronic invasive nongranulomatous FRS should be kept in mind in the presence of long-standing nonspecific sinonasal symptoms in immunocompetent individuals with a history of working in greenhouse farming.

## Figures and Tables

**Table 1:** Characteristics and outcome of the patients.

	Case 1	Case 2	Case 3	Case 4
Age/gender	58 yrs/male	43 yrs/male	60 yrs/female	68 yrs/male
Immune suppression	−	−	−	−
Symptom onset to diagnosis	6 months	24 months	9 months	6 months
Symptoms at presentation	Left orbital pain	Nasal obstruction	Dizziness	Headache
	Swelling on the face	Headache	Headache	Left retroorbital pain
	Nasal obstruction	Facial pain	Postnasal drip	
Anatomic subsite involvement				
Nasal cavity	+	+	−	−
Osteomeatal complex	+	+	−	−
Maxillary sinus	+	−	−	−
Ethmoidal sinuses	+	+	+ (right posterior)	+ (right posterior)
Sphenoid sinus	+ (left side)	+ (left side)	+ (right side)	+ (right side)
Frontal sinus	+	+	−	−
Bilateral involvement	−	−	−	−
Orbital involvement	LP destruction	LP destruction	−	−
Intracranial extension	−	−	+	−
Surgical treatment	ESS	ESS combined with external frontal sinus surgery	ESS	ESS
Organism				
Microbiology	*A. fumigatus*	*A. flavus*	*A. fumigatus*	−
Histopathology	Nongranulomatous CIFRS	Nongranulomatous CIFRS	Nongranulomatous CIFRS	Nongranulomatous CIFRS
Medical treatment				
Voriconazole	+	+	+	+
Follow-up time	10 months	8 months	7 months	5 months
Disease recurrence	−	−	−	−

LP: lamina papyracea, ESS: endoscopic sinus surgery, and CIFRS: chronic invasive fungal rhinosinusitis.
